# Identifying effective factors to alleviate postnatal distress and coronavirus anxiety in mothers of hospitalized preterm neonates

**DOI:** 10.1186/s12884-023-06131-1

**Published:** 2023-12-06

**Authors:** Borghei Narjes Sadat, Mehrbakhsh Zahra, Torklalebaq Fatemeh

**Affiliations:** 1 Reproductive Health, Counseling and Reproductive Health Research Center, School of Nursing and Midwifery, Golestan University of Medical Sciences, Gorgan, Iran; 2 Department of Biostatistics, School of Public Health, Hamadan University of Medical Sciences, Hamadan, Iran; 3 Department of Biostatistics and Epidemiology, School of Health, Golestan University of Medical sciences, Gorgan, Iran; 4 Faculty of Nursing and Midwifery, Pregnancy Health Research Center, Zahedan University of Medical Sciences, Zahedan, Iran

**Keywords:** Anxiety, COVID-19, Postnatal distress, Prematurity

## Abstract

**Background:**

Given the critical importance of mental health in mothers of preterm neonates during the postpartum period for Population Youth Programs, our research aims to ascertain the correlation between postnatal distress and corona-induced anxiety in women who have hospitalized preterm neonates.

**Methods:**

This descriptive-analytical study was conducted with a sample of 275 mothers of preterm neonates, were hospitalized in Gorgan city in 2020. Data collection was facilitated through the Corona Anxiety (CA) and Postnatal Distress Measured Scale (PDM). For data analysis, Spearman’s correlation and univariate and multiple linear regression were employed.

**Results:**

The average age of the participating mothers was 28.61 ± 6.173 years, and the average gestational age of the neonates was 32.8 ± 2.89 weeks. The study found a significant, positive correlation between CA and PDM. Controlling for other variables through multiple regression analysis, the factors that significantly influenced PDM were employment status (*β = 3.88, p < 0.01*), education level (*β = 1.96, p = 0.032*), and gestational age (*β=-0.60, p < 0.001*). Furthermore, number of living children (*β=-4.77, p = 0.01*), education (*β=-2.37, p = 0.01*), and gestational age (*β=-0.91, p < 0.001*) were the factors that were significantly associated with CA scores.

**Conclusions:**

The correlation between CA and PDM suggests that preterm neonate’s mothers experienced increased anxiety during the pandemic. Considering the factors influencing these anxieties, targeted programs should be developed to enhance the mental health of these mothers in future pandemics. The finding that women with more children experienced less CA could serve as evidence of the positive impact of having children on the mental health of women with premature infants during a pandemic.

## Background

Parents, who anticipate the birth of a healthy newborn for months, experience profound shock and stress when confronted with the birth of preterm infants requiring hospitalization and connection to medical equipment [1–3]. Parents of preterm infants often experience heightened anxiety compared to those with full-term, healthy newborns [1, 4–6]. Notably, mothers’ anxiety levels tend to be higher than fathers’ [5], making mothers of hospitalized preterm infants particularly susceptible to depression and anxiety, conditions that often go overlooked [7, 8]. In 2015, the World Health Organization (WHO) reported that the rate of preterm birth across 184 countries ranged from 5 to 18% [9]. Bayat Mokhtari et al. (2009) reported a preterm birth rate of 6% in Mashhad [10], while Tabandeh and Kashani (2007) reported a rate of 9.42% at Dezyani Hospital-Gorgan in a cross-sectional analytical study [11]. According to research by Younger et al. (Virginia State, 1997), between 28 and 71% of mothers with hospitalized preterm infants suffered from severe anxiety [12]. Banner’s research in Qatar in 2013 reported that the level of anxiety among these mothers was 32% [13].

The anxiety experienced by mothers is associated with diminished maternal affection and responsibility, and disruptions in parental behavior [14–16], which negatively affect mother-infant bonding [17], the infant’s emotional and physical development [18], and even increase the risk of exacerbated mental health issues in mothers during and after the hospitalization of preterm infants [16, 18]. Post-traumatic stress disorder is also more prevalent in mothers of hospitalized preterm infants than in mothers of healthy newborns [5, 19, 20]. Approximately 34% of mothers with hospitalized preterm infants experience suicidal thoughts, a significantly higher percentage compared to the 14% reported by other mothers [21, 22]. The anxiety levels of mothers do not depend on the severity of the infant’s prematurity or socio-economic status but are correlated with the mother’s level of education and her social support system [15]. Moreover, reducing anxiety in these vulnerable mothers can prevent numerous anxiety-related complications in both mothers and infants [23].

 The advent of the COVID-19 pandemic has amplified the stress and anxiety of pregnant women worldwide [24]. Most pregnant women experienced severe anxiety about contracting the coronavirus and potentially transmitting it to their fetuses [25]. Sometimes, this fear even led them to avoid medical appointments or opt for early termination and elective caesarean Sect. [26]. These anxieties often carried over into the postpartum period [27], adding an extra layer of stress for women with preterm infants during the COVID-19 pandemic [24]. Several studies have explored pregnancy and coronavirus-related anxiety, such as the research by Alizadeh-Fard and Saffarinia (2009). They reported that coronavirus-induced anxiety (negatively) and the sense of social solidarity spurred by the pandemic (positively) were correlated with mental health. Furthermore, they found that anxiety and social solidarity due to the pandemic predicted 47% and 26% of mental health changes, respectively [28]. Zolfaghari and Elahi reported a significant correlation between children’s anxiety levels and their mothers’ anxiety, stress, and depression, as well as the children’s awareness of the coronavirus and their age. Moreover, they found that mothers’ anxiety, depression, and stress, along with children’s awareness of the coronavirus, were significant predictors of children’s anxiety regarding the coronavirus disease crisis (accounting for a total of 34%) [29]. However, the researcher did not identify any studies examining the correlation between coronavirus-induced anxiety and postnatal distress.

In light of the COVID-19 pandemic and a noticeable absence of published studies on coronavirus anxiety and postnatal distress in Iranian postpartum women, coupled with a limited amount of research on the anxiety experienced by mothers with hospitalized preterm neonates during the pandemic, the researchers aimed to identify the factors that could effectively improve postnatal distress and alleviate coronavirus anxiety. By doing so, they hoped to contribute to enhancing the mental health of postpartum mothers with preterm neonates using the insights gained from their research.

## Methods

### Aim, design and setting of study

This study was conducted in in Gorgan City, central Golestan province in north-east of Iran, located in south of the Caspian Sea (36° 50′ 21.48″ N, 54° 26′ 39.84″ E).

This cross-sectional, descriptive-analytical study was conducted to examine 275 mothers with hospitalized preterm neonates during the second wave of COVID-19 in 2020, with aim to investigate the correlation between postnatal distress and coronavirus anxiety. The research was carried out in all hospitals in Gorgan that feature neonatal intensive care units, including Sayad Shirazi, Taleghani, and Hakim Jorjani Hospitals.

Participants were chosen through convenience sampling. Following their inclusion in the study, and after receiving an explanation of the research purpose, eligible mothers who signed informed consent, completed demographic information forms, the coronavirus anxiety scale, and the postnatal distress measure scale as self-reports.

### The characteristics of participants or description of materials

The inclusion criteria were as follows: the mother’s ability to read and write, absence of neonatal anomalies, and evidence of neonatal prematurity (gestational age less than 38 weeks, as approved by a pediatrician’s clinical examination and recorded in the neonate’s file). The exclusion criteria included the mother’s use of sedatives and hypnotics, having a sick neonate, experiencing a traumatic event in the past six months such as the death of a loved one, and the husband’s engagement in polygamy.

### Sample size

According to one of the purpose of the research, to determine the correlation between Corona anxiety [30] and distress after childbirth [31], the relationship between anxiety and distress was used because the Corona anxiety questionnaire was designed and validated during the Corona outbreak and similar studies could not be found for this reason.

Assuming that there is no linear relationship between anxiety and distress (the correlation coefficient between anxiety and distress is zero), the sample size is estimated to be 26 people.

To determine the maximum sample size, considering the hypothesis that there is a linear relationship between the two variables, and considering the correlation coefficient (0.4) in the null hypothesis, with a confidence level of 95% and a power test of 80%, a total sample size of 275 was calculated.

$$n={\left(\frac{{Z}_{{}^{\alpha }\!\left/ \!{}_{2}\right.}+{Z}_{\beta }}{{C}_{1}-{C}_{2}}\right)}^{2}+3=275$$

$${C}_{1}=\frac{1}{2} ln\left[\frac{1+{r}_{1}}{1-{r}_{1}}\right]=\frac{1}{2} ln\left[\frac{1+0.53}{1-0.53}\right]=0.59$$

$${C}_{2}=\frac{1}{2} ln\left[\frac{1+{r}_{2}}{1-{r}_{2}}\right]=\frac{1}{2} ln\left[\frac{1+0.4}{1-0.4}\right]=0.42$$

Also, the number of samples required to fit the appropriate regression model is at least 5 samples for each variable. Based on the sample size determined, the minimum sample size required to fit the regression model is available.

### Data collection

Three tools were utilized to collect data in this study:

1. A demographic information checklist was used to collect details about age, ethnicity, education level, employment, economic status, history of pregnancy, abortions, number of live children, and history of infertility. This information was provided by the participants themselves.
2. The Coronavirus Anxiety Scale, validated and approved by Alipour et al. (2020), was used. This scale, completed by 308 participants in an online survey, consists of 18 questions divided into two parts on a four-point Likert scale. The first nine items measure psychological symptoms and the remaining nine measure physical symptoms. Scores range from 0 to 54, with higher scores indicating greater anxiety. The total score can be classified into three categories: mild (0–16), moderate (17-29), and severe (30–54). The internal consistency of the items was confirmed by measuring Cronbach’s alpha, with coefficients obtained for psychological symptoms (0.87), physical symptoms (0.86), and the entire questionnaire (0.91) [30].
3. The Postnatal Distress Measure (PDM) Scale, initially designed and validated by Allison et al. in 2011 [32] and later approved for use during pregnancy in 2017 [33], was also employed. The Persian version of this tool was psychometrically assessed by Shokouhi et al. (2020). The validity of the tool was tested through face, content, and construct stages with the help of 10 knowledgeable experts and 150 mothers who attended health centers for postpartum care. Concurrent validation was conducted using the Depression, Anxiety, and Stress Scale- 21 Items (DASS-21). The tool’s reliability was confirmed by measuring Cronbach’s alpha (0.72). The content validity index (0.94) and impact score (2.97) also affirmed the tool’s validity. The PDM scale consists of ten items scored from 0 to 3. Items 1, 6, and 10 are scored directly, while items 2, 3, 4, 5, 7, 8, and 9 are scored in reverse. Scores range from 0 to 30, with higher distress reflected in higher scores [31].

### Statistical method

The data was analyzed using SPSS 16. Mean and standard deviation were employed to describe quantitative variables, while frequency and percentage were utilized for qualitative variables. The normality of the quantitative variables was assessed through the Shapiro-Wilk test. The correlation between coronavirus anxiety and postnatal distress was evaluated using Spearman’s correlation test. Both univariate and multiple linear regression models were applied to explore the impact of demographic and fertility variables on coronavirus anxiety and postnatal distress. The significance level for the tests was set at 0.05.

### Ethical approval

In conducting this study, all ethical considerations were meticulously observed. These include obtaining a sampling permit, ensuring the confidentiality of information, and procuring informed consent. The ethical code of this project (IR.GOUMS.REC.1399.334) was duly registered on the website of the National Committee for Ethics in Biomedical Research.

## Results

The study encompassed 275 mothers, who had an average age of 28.61 years with a standard deviation of 6.17. The average gestational age was 32.8 weeks, within a range of 26–37 weeks, and a standard deviation of 2.89. As illustrated in Table 1, a majority of the women from Fars were unemployed, possessed a medium-level economic status, were highly educated, and had experienced three or more pregnancies (Table 1).

**Table 1:** Demographic and fertility characteristics of women

Variables	Number	percent
Employment status		
Not Employed	229	83.30
Employed	46	16.70
Economic status		
Weak	110	40
Medium	120	43.60
Good	45	16.40
Literacy		
Low	87	31.60
Diploma	75	27.30
High	113	41.10
Ethnicity		
Fars	130	47.30
Turkmen	93	33.80
Sistani	42	15.30
^a^other	10	3.60
Gravid		
1	84	30.50
2	90	32.70
More than 3	101	36.80
Living child		
1	105	38.20
2	112	40.70
More than 3	58	21.10
Infertility history		
No	257	93.50
Yes	18	6.50
Abortion history		
No	190	69.10
Yes	85	30.90

^a^Other: tork, kormanj. Zaboly

The results of the Coronavirus Anxiety Scale showed a range of 10 to 38, with mean score 21.60(SD = 6.73). Additionally, the scores on the PDM Scale ranged from 0 to 30, with mean score10.65 (SD = 5.86). The research results presented a positive and statistically significant correlation between coronavirus anxiety and postnatal distress, with a correlation coefficient (r) of 0.38.

To identify the factors contributing to coronavirus anxiety and postnatal distress, both univariate and multiple linear regression models were applied. The regression model incorporated job status, economic status, and ethnicity variables in the univariate linear regression model. The education level and gestational age variables were included in both univariate and multiple linear regression models. Furthermore, the number of living children was considered a significant predictive factor for coronavirus anxiety in the multiple regression model.

Univariate and multiple linear regression models were applied to identify the significant factors influencing coronavirus anxiety and postnatal distress. The regression model incorporated job status, economic status, and ethnicity as variables in the univariate linear regression model. In both univariate and multiple linear regression models, education level and gestational age were included. The number of living children was significant in the multiple regression model and was hence considered a predictive factor for coronavirus anxiety.

In a multiple regression model, with other variables controlled, the number of living children had the most substantial impact on coronavirus anxiety (*β=-4.77, p = 0.01*), followed by education level (*β=-2.37, p = 0.01*) and gestational age (*β=-0.91, p < 0.001*). Women with more than three children displayed anxiety scores that were 5 units lower than those of women with a single child. Moreover, women with high education levels exhibited anxiety scores approximately 2.5 units lower than those of women with lower education levels. Each additional week of gestational age corresponded to a one-unit decrease in the coronavirus anxiety score (Table 2).

**Table 2:** The effect of demographic and fertility variables on corona anxiety using univariate and multiple linear regression

Variable	Univariate Linear Regression		Multiple Linear Regression	
	Coefficient estimates (Std. Error)	*P*-value	Coefficient estimates (Std. Error)	*P*-value
Employment status				
Not Employed	Reference			
Employed	− 2.73(1.07)	0.01	-	
Economic status				
Weak	Reference			
Medium	1.14(0.88)	0.19	-	
Good	2.50(1.18)	0.03	-	
Literacy				
Low	Reference		− 1.30(0.98)	0.18
Diploma	-0.83(1.04)	0.42		
High	− 3.07(0.94)	0.001	− 2.37(1.00)	0.01
Ethnicity				
Fars	-7.33(2.16)	0.001	-	
Turkmen	− 6.71(2.19)	0.002	-	
Sistani	− 5.00(2.32)	0.03	-	
^a^other	Reference			
Gravid				
1	Reference			
2	− 0.29(1.025)	0.77	-	
More than 3	− 0.33 (0.99)	0.73	-	
Living child				
1	Reference			
2	− 0.53(0.91)	0.56	− 1.37(1.28)	0.28
More than 3	− 1.4 (1.10)	0.20	- 4.77(1.88)	0.01
Infertility history				
No	Reference			
Yes	− 2.90(1.63)	0.07	-	
Abortion history				
No	Reference			
Yes	- 0.17(0.88)	0.84	-	
GA				
-	− 0.93 (0.12)	< 0.001	- 0.91(0.12)	<0.001

^a^Other: tork, kormanj. Zaboly

In both univariate and multiple linear regression models, job status, education level, and gestational age were considered the predictive factors of postnatal distress. Through multiple regression and controlling other variables, job status (*β = 3.88, p < 0.01*), education level (*β = 1.96, p = 0.032*), and gestational age (*β=-0.60, p < 0.001*) exerted the most substantial influence on postnatal distress. As such, employed women experienced greater postnatal distress than unemployed women. Similarly, women with diplomas experienced more distress than those with lower educational attainment. Additionally, each week’s increase in gestational age reduced the postnatal distress score by nearly half (Table 3).

**Table 3:** The effect of demographic and fertility variables on postpartum distress using univariate and multiple linear regression

Variable	Univariate Linear Regression		Multiple Linear Regression	
	Coefficient estimates (Std. Error)	*P*-value	Coefficient estimates (Std. Error)	*P*-value
Employment status				
Not Employed	Reference			
Employed	2.60(0.93)	0.006	3.88(1.05)	<0.001
Economic status				
Weak	Reference			
Medium	0.56(0.77)	0.47	-	
Good	0.22(1.04)	0.82	-	
Literacy				
Low	Reference		1.96(0.91)	0.032
Diploma	1.32(0.92)	0.15		
High	1.47(0.83)	0.07	1.21(0.92)	0.19
Ethnicity				
Fars	-0.69(1.91)	0.71	-	
Turkmen	− 1.91(1.93)	0.32	-	
Sistani	0.85(2.04)	0.67	-	
^a^other	Reference			
Gravid				
1	Reference			
2	− 0.82(0.88)	0.35	-	
More than 3	0.41 (0.86)	0.63	-	
Living child				
1	Reference			
2	− 0.41(0.79)	0.60	-	
More than 3	− 0.21 (0.96)	0.82	-	
Infertility history				
No	Reference			
Yes	− 0.99(1.43)	0.48	-	
Abortion history				
No	Reference			
Yes	1.33(0.76)	0.08	-	
GA				
-	− 0.51 (0.11)	< 0.001	- 0.60(0.11)	< 0.001

^a^Other: tork, kormanj. Zaboly

## Discussion

According to recent studies, women have been more affected by psychological distress than men, and gender plays a significant role in the psychological consequences of COVID-19 [34]. Women exhibit higher symptoms of coronavirus-related anxiety than men do [35]. It is well understood that anxiety and stress tend to increase during pregnancy. A study conducted in Pakistan revealed that 70% of pregnant women experienced anxiety and depression [36], with most of these anxieties persisting into the postpartum period.

Research on prenatal and postnatal distress among American and Canadian pregnant women found that out of 288 participants, 21.2% experienced prenatal mental distress, while 70.5% faced postnatal distress. The study concluded that prenatal distress is a predictor of postpartum stress, anxiety, and depression [27].

Post-birth, mothers undergo various types of stress. An Australian study segmented postpartum distress into four phases: behavioral (encompassing the mother’s sleep-relief), cognitive (highlighting uncertainty and shared human experience), child-related (focusing on sleep, nutrition-growth, and development), and social (including support) [37]. Mothers with preterm neonates tend to experience greater postnatal distress, particularly concerning neonate care [38].

The advent of the COVID-19 pandemic has exacerbated this distress, particularly for mothers with hospitalized preterm neonates. As a result, pregnancy has been linked with increased anxiety and stress during the COVID-19 pandemic [24], occasionally leading to adverse outcomes [39]. A Pakistani study reported that most pregnant women suffered heightened stress over the risk of contracting COVID-19 and transmitting it to their neonates [25].

In the study presented here, a moderate correlation was observed between postnatal distress and coronavirus anxiety. However, the reported levels of anxiety varied among different ethnic groups, with lower rates typically reported among Asians, likely due to their unique ethnic and cultural beliefs [35]. Research involving 900 Canadian postpartum women revealed that 29% had moderate to high anxiety (anxiety score > 40 in positional and dispositional anxiety) prior to the pandemic, a figure that rose to 72% following the pandemic’s onset [40]. Another Canadian study also confirmed an uptick in the level and intensity of postpartum anxiety during the COVID-19 pandemic [41].

A cross-sectional study conducted in Belgium involving 3445 postpartum women found that approximately 42% exhibited symptoms of anxiety during the COVID-19 quarantine [42]. Contrastingly, a Danish study reported no correlation between anxiety and postpartum stress before and after the COVID-19 pandemic among nulliparous women [43].

Prenatal anxiety and stress are known to increase complications such as preterm birth [44]. There is a direct correlation between the anxiety levels of mothers and their children [45], and an inverse correlation between anxiety and mental health [46]. Consequently, there is a pressing need for effective interventions to reduce anxiety and stress during the pandemic, to prevent their subsequent repercussions [47].

In addition, it was observed that maternal anxiety and distress declined as gestational age increased, suggesting that effective training and care aimed at preventing preterm birth could significantly enhance the mental health of postpartum women. Cultivating trust between healthcare personnel and mothers [37] and the perception of support delivered by healthcare workers [48] are critical strategies in this respect. It is recommended that families undergo training to bolster social support and to maintain the accompanying midwife support program (doula) at least until two months post-birth.

Moreover, the number of living children had the greatest influence on coronavirus anxiety. Women with more than three children exhibited lower coronavirus anxiety due to the fear of child loss prevalent among nulliparous mothers [49]. Having more live births can mitigate the anxiety about losing this index preterm birth, although what is unclear, and not examined in this study is whether there are other concerns about the well-being of the other children.

The current study’s findings also indicate that postnatal distress was more pronounced among highly-educated and employed mothers. A study on postnatal distress in mothers with preterm infants showed increased levels of anxiety and depression. However, this anxiety was less evident in women with lower educational attainment [13]. Mothers who are highly educated and employed likely absorb more information from their surroundings, which may contribute to heightened anxiety. Additionally, the limited time a working mother spends with her child can trigger anxiety in the child [45]. Also, it should be noted that working mothers would be anxious about getting infected during the pandemic as it is possible that these were essential workers who needed work on-site, and therefore be exposed to the virus, and have anxieties about bringing the infection back to their families, especially the vulnerable preterm baby.

Therefore, it may be feasible to reduce postnatal distress by developing supportive policies for working mothers and should be to advocate to safe working measure particularly for mothers who have delivered preterm, in the critical situation of a disease pandemic.

The current study found that ethnicity did not significantly influence postnatal anxiety and distress levels, but Brotto et al. examined the correlation between coronavirus anxiety and ethnic and cultural factors in Canada, revealing that Chinese and Taiwanese ethnic groups had fewer symptoms of coronavirus anxiety, while Canadian natives exhibited more symptoms during the COVID-19 pandemic [35]. A study in Japan reported similar rates of postpartum depression before and after the pandemic [50]. A comparison of the anxiety levels between Arab and Jewish women revealed that although coronavirus anxiety was prevalent among pregnant women, Arab women were more anxious than Jewish women [51]. All ethnicities investigated in the present study were Asian and it appears that Asians reported less anxiety, likely due to their ethnic and cultural beliefs [35].

## Research strengths, weaknesses and limitations

The research sampling took place during the peak of the COVID-19 pandemic’s second wave, providing valuable and challenging-to-obtain information for researchers. However, due to the sensitive circumstances surrounding hospitalized preterm neonates during the pandemic and medical complications related to the preterm birth as a mother with an ill infant will have more heightened anxiety, thus these mothers were often reluctant to participate in the research. As a result, the sampling process spanned six months (summer and autumn 2020).

## Conclusion

Pregnancy often leads to heightened anxiety. Consequently, women with preterm neonates tend to be more engaged in their care, resulting in increased postpartum distress. Premature birth is one of the consequences of anxiety, which increased significantly during the corona pandemic. There is a moderate correlation between postpartum distress and anxiety related to the coronavirus pandemic, suggesting that mothers’ anxiety levels have risen during this period. Given the factors that can alleviate these anxieties, structured programs are essential to reduce postpartum distress, ensuring that their mental health is not overlooked in future pandemics.

## Data Availability

The datasets used and analyzed during the current study available from the corresponding author on reasonable request.
